# Bio-Inspired Magnetically Responsive Silicone Cilia: Fabrication Strategy and Interaction with Biological Mucus

**DOI:** 10.3390/bioengineering11030261

**Published:** 2024-03-07

**Authors:** Aline Grein-Iankovski, Karina Andrighetti de Oliveira Braga, Daniel Formariz Legendre, Paulo Francisco Guerreiro Cardoso, Watson Loh

**Affiliations:** 1 Institute of Chemistry, Universidade Estadual de Campinas (UNICAMP), Campinas 13083-970, SP, Brazil; agrein@unicamp.br; 2 Instituto do Coração, Hospital das Clinicas HCFMUSP, Faculdade de Medicina, Universidade de São Paulo, São Paulo 01246-903, SP, Brazil; kariandri@usp.br; 3 Fundação Adib Jatene, Instituto Dante Pazzanese de Cardiologia, São Paulo 04012-180, SP, Brazil; daniel@fajbio.com.br

**Keywords:** magnetic cilia, artificial cilia, silicone, tracheal prosthesis, mucociliary clearance

## Abstract

Cilia are biological structures essential to drive the mobility of secretions and maintain the proper function of the respiratory airways. However, this motile self-cleaning process is significantly compromised in the presence of silicone tracheal prosthesis, leading to biofilm growth and impeding effective treatment. To address this challenge and enhance the performance of these devices, we propose the fabrication of magnetic silicone cilia, with the prospect of their integration onto silicone prostheses. The present study presents a fabrication method based on magnetic self-assembly and assesses the interaction behavior of the cilia array with biological mucus. This protocol allows for the customization of cilia dimensions across a wide range of aspect ratios (from 6 to 85) and array densities (from 10 to 80 cilia/mm^2^) by adjusting the fabrication parameters, offering flexibility for adjustments according to their required characteristics. Furthermore, we evaluated the suitability of different cilia arrays for biomedical applications by analyzing their interaction with bullfrog mucus, simulating the airways environment. Our findings demonstrate that the fabricated cilia are mechanically resistant to the viscous fluid and still exhibit controlled movement under the influence of an external moving magnet. A correlation between cilia dimensions and mucus wettability profile suggests a potential role in facilitating mucus depuration, paving the way for further advancements aimed at enhancing the performance of silicone prostheses in clinical settings.

## 1. Introduction

The human trachea and bronchi are complex structures designed to carry the airflow in and out of the lungs and to mobilize secretions from the lungs and bronchi outwards. The inner airway epithelium has moving microcilia that act as a self-cleaning apparatus to move secretions and particulate matter from the lower airway towards the larynx, where they may be swallowed or coughed out [1]. Such cleaning properties are impaired by congenital and acquired diseases that affect the mobility of the cilia [2].

Tracheal stenosis is the segmental airway narrowing resulting from congenital and acquired benign or malignant airway diseases. The most prevalent cause of tracheal stenosis is the segmental scarring of the airway caused by intubation or tracheostomy used for mechanical ventilatory assistance. The reported incidence of post-intubation tracheal stenosis varies from 0.6% to 21% [3]. The recent COVID-19 pandemic caused a significant rise in post-intubation airway stenosis due to the high number of patients with Severe Acute Respiratory Syndrome Coronavirus 2 lung disease (SARS-CoV-2) requiring prolonged mechanical ventilatory support worldwide [4].

The local contributing factors for the occurrence of airway stenosis include inner airway trauma deriving from the contact between the tracheal wall and the endotracheal or tracheostomy tube, inadequate management of the cuff pressure, technical problems during the tracheostomy, and changes in the tracheal mucosal bacterial proliferation. Other contributing factors such as cardiovascular disease, diabetes, infections, and gastroesophageal reflux [5] can also contribute to the onset, severity, and recalcitrant character of tracheal stenosis [6].

Patients with tracheal stenosis may require prolonged stenting to maintain the airway patency. Airway silicone stents are indwelling prostheses often used for long-term stenting of tracheal stenosis. These tubular stents come in different diameters and shapes to fit into the airway lumen. The stents are placed under endoscopic control through the previously dilated stenosis to prevent narrowing and airway obstruction during healing. Silicone airway prostheses are designed to stay in place from several months to a few years, depending on the extent and nature of the disease process, ensuring patency while the tracheal disease process subsides and the trachea heals. The stents are also used as a bridge when local or systemic conditions do not allow for definitive surgical removal of a diseased airway segment and primary reconstruction.

The local contact area of the silicone prosthesis’ surface with the airway mucosa interferes with the airway epithelium mucous clearance of both secretions and particulate matter. Furthermore, the constant contact with the external milieu through breathing develops an endoluminal multi-microbial biofilm in the stent’s inner and outer silicone surface. It interacts with the adjacent tracheal mucosa, thus interfering with the proper healing of the disease process and causing a negative quality of life for the patient [7]. The performance of silicone tracheal prosthesis depends on its self-cleaning capability, which relies solely on the patient’s cough and the use of nebulized aerosols. We hypothesized that the presence of acting movable biomimetic ciliated structures applied to the inner surface of the silicone prosthesis could reduce biofouling by moving secretions and reducing bacterial adhesion to the biofilm, thus helping to improve treatment outcomes of patients with tracheal stenosis wearing silicone airway prosthesis.

Over the last decade, researchers have been engineering artificial cilia to mimic the functions of biological cilia for various applications, including microsensors, microrobots, microfluidic mixing, and self-cleaning and antifouling surfaces [8]. Among the materials explored for fabricating these structures and the strategies employed for inducing motion in the ciliary arrays, the use of magnetic particles is particularly noteworthy [9]. These artificial cilia, embedded with magnetic particles, can be remotely activated by applying an external magnetic field, enabling contactless motion [8,10]. Different strategies for fabricating magnetic cilia have been developed, generally classified into molding [11,12,13,14,15,16] or self-assembly [17,18,19,20,21,22,23,24,25] processes.

Our approach is based on a magneto-driven self-assembly approach, chosen for its adaptability and efficiency in scaling up production. However, a significant drawback of this method is the difficulty in achieving precise uniformity in the position and size of the pillars compared to molding techniques. As a result, there is typically a variation of 10% to 40% in the dimensions of the cilia [18,19,21]. Despite this limitation, the self-assembly approach offers the advantage of achieving higher aspect ratios and involves a simpler manufacturing process [26]. Previously, we described a method for the fabrication of magnetic cilia using a thermoresponsive composition, employing a similar self-assembly approach [27,28]. This method served as the foundation for producing elastomeric silicone magnetic cilia for application in silicone airway prostheses.

The present paper describes the process for the fabrication of a variety of magnetically responsive silicone cilia deposited onto a silicone substrate, displaying varied aspect ratios and surface densities. By carefully controlling the fabrication parameters, it is possible to tailor the cilia dimensions within a variation of approximately 10% amongst independent replicates. For applications that do not require a strict uniformity of the cilia sizes, the current fabrication method has advantages for industrial scalability, considering its low cost, preparation time, and simplified manufacturing. We also assessed the interaction of cilia-covered surfaces with biological mucus in a preliminary study which showed an interdependent effect of the cilia characteristics with the organic mucus deposition profile and potential depuration.

## 2. Experimental Section

### 2.1. Fabrication of Magnetic Cilia

The magnetic cilia samples were fabricated based on a template-free magneto-driven self-assembly approach using polydimethylsiloxane (DOWSIL^TM^ EI-1184, Dow Chemical Company, Midland, MI, United States) and magnetic carbonyl iron particles (average diameter 2.0 ± 0.7 µm, CIP-EW, BASF, Ludwigshafen, Germany) as starting materials. Initially, stock solutions of polydimethylsiloxane (PDMS) components Part A and Part B, referring to base polymer and curing agent compositions, were separately prepared in ethyl acetate at 15 wt% and 30 wt%, respectively. Just before the cilia fabrication, the PDMS components A and B were diluted in ethyl acetate if necessary and mixed at a mass ratio of 1:2. The amount of PDMS used in each sample was determined relative to the mass of magnetic particles (MP) added, ensuring a constant MP to PDMS (Parts A + B) mass ratio of 1:10 in all samples (omitting solvent). The amounts of magnetic particles added varied among 0.6, 0.9, 1.2, and 1.8 mg. The MP dispersion in PDMS was homogenized by sonication for 2 min in a 40 kHz ultrasonic bath (Branson CPX1800H, Emerson, St. Louis, MO, United States). Subsequently, 80 µL of this dispersion was transferred to a polytetrafluoroethylene (PTFE) vessel with an internal diameter of 8 mm, containing a cured PDMS disc (1 mm thick) as the substrate for cilia growth. The vessel was immediately placed over a permanent NdFeB magnet (3.0 cm diameter, 1.0 cm thickness, 0.3 T). The distance from the sample to the magnet varied among 0.1 cm (close contact with the substrate), 0.7 cm, and 1.2 cm. The magnetic particles quickly aligned in the dispersion under the influence of the magnetic field, forming elongated structures perpendicular to the base. As the solvent evaporated, the PDMS formed a film on the substrate, trapping the protruding MP pillars within the polymer matrix. The PDMS elastomer was then cured at 65 °C for 48 h, resulting in durable and flexible cilia structures. Upon removing the sample from the container, any excess material adhered to the substrate’s edges was trimmed using a scalpel.

### 2.2. Structural Characterization of the Cilia

The characterization of the cilia dimensions according to the fabrication parameters was performed using optical microscopy with a Nikon Eclipse 50i microscope. Measurements of cilia length, thickness at half-length, and surface density (number of cilia per surface area) were carried out using ImageJ software. Each data point represents the combined mean and standard deviation [29] of two independent replicates, considering the measurements of at least 100 objects per sample. The surface morphology of the cilia and the distribution pattern of magnetic particles within the PDMS matrix were examined using scanning electron microscopy (SEM) with a FEI Quanta FEG 250 microscope equipped with a secondary electron Everhart–Thornley detector (ETD) and a circular backscatter electron detector (CBS). The accelerating voltage was varied from 5 to 30 kV. For SEM analyses, one sample was trimmed at half-length to analyze the cilia cross-section, and some cilia were cut out from the substrate at the base and deposited onto carbon tape.

### 2.3. Qualitative Analysis of Cilia Interaction with Mucus

In an in-vitro preliminary study, fabricated cilia samples of varying dimensions were subjected to the deposition of fresh mucus obtained from bullfrogs (*Rana catesbeiana*). These cilia samples were exposed to a nebulized environment with an air humidity ranging from 90% to 95%. A drop of the biological mucus was then placed over the cilia array. The interaction behavior and wettability profile of each sample were analyzed using an optical microscope. Additionally, the susceptibility of the cilia to motion induced by manual sliding actuation of a magnet underneath and parallel to the surface was examined. The qualitative assessment of the mucus–cilia interaction included an analysis of the wettability profile, mechanical resistance of the cilia structures, and their motion capacity upon contact with the viscous biological fluid. The animal experiments were performed in compliance with the Ethics Committee for Experimental Animals Use and Care of the Faculty of Medicine of the University of São Paulo (protocol code 2072/2024).

## 3. Results and Discussion

### 3.1. Fabrication of the Magnetic Cilia

**Figure 1:**
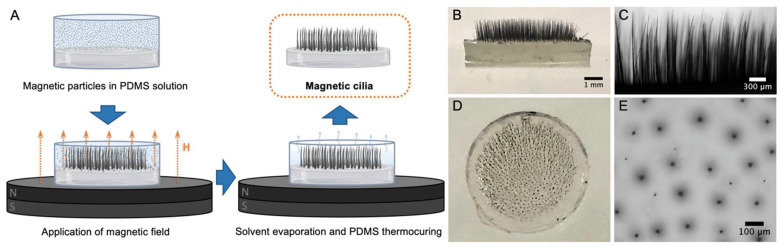
(**A**). Illustration of the cilia preparation procedure. Pictures and optical microscopy images of the cilia fabricated over a silicone disc were taken from lateral (**B**,**C**) and downward views (**D**,**E**).

A schematic representation of the fabrication procedure is depicted in Figure 1A. The dispersion, consisting of magnetic particles (MP) and polydimethylsiloxane (PDMS) mixture, was poured over a cured PDMS disc and quickly centered within the magnetic field. The MPs promptly distribute over the surface and align vertically throughout the dispersion, following the magnetic flux lines that act as propulsion for cilia growth [30]. As the solvent evaporates, the PDMS chains entrap and deposit over the aligned MP, forming the cilium body. Subsequent thermal curing results in a reticulated PDMS mesh, providing mechanical stability and flexibility for the cilia structures, while the magnetic particles enable motion susceptibility for actuation with an external magnetic field. The resulting structures are illustrated in Figure 1B,D (pictures) and Figure 1C,E (optical microscopy images), showcasing lateral and downward views, respectively. Video S1 (Supplementary Material) demonstrates the flexibility of the fabricated cilia and their susceptibility to magnetic motion. The cilium structures exhibit a random distribution over the silicone surface, maintaining a certain distance between them, and can expand upward to varying heights depending on the fabrication conditions.

### 3.2. Characterization of Magnetic Cilia

**Figure 2:**
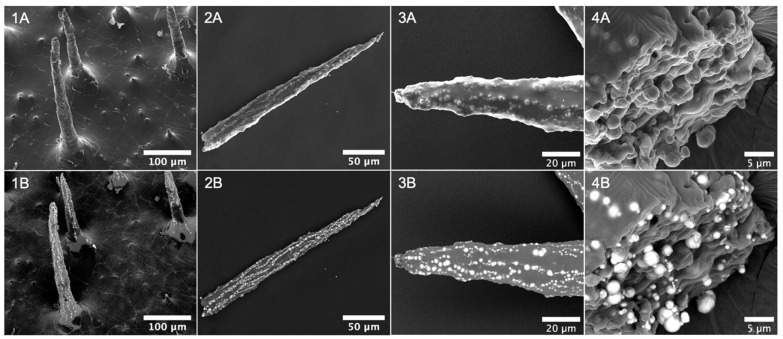
SEM images of downward view of cilia distribution with trimmed tips (**1A**,**1B**), lateral view of a cilium (**2A**,**2B**), the morphology of the cilium upper extremity (**3A**,**3B**), and cross-section cut of a cilium (**4A**,**4B**). Top images (labels **A**) were obtained with a secondary electrons detector (ETD) and showed surface topographic information. Bottom images (labels **B**) represent the same view captured with a circular backscatter detector (CBS), enhancing contrast dependence on materials composition, and highlighting the MP as bright domains. Accelerating voltage of 10 kV.

The structures and morphology of the produced cilia were analyzed using scanning electron microscopy (SEM), as depicted in Figure 2. Figure 2(1A,1B) provides detailed views of the cilia attachment on the PDMS base, revealing that magnetic particles (MP) are primarily concentrated within the cilia-protruding structures, with the PDMS body extending as a film over the base surface between them. The distribution of these structures across the surface is non-uniform, with the distance between the cilia varying from approximately 110 to 230 µm within the observed sample region. These images also clearly demonstrate the vertical alignment of the MP along the length of the pillar, which is maintained after the removal of the magnet. Capillary force drives the resulting elongated conical shape, as depicted in Figure 2(2A,2B), gradually forming a sharper tip until reaching a constant value [31,32]. In this example, the cilium length measures approximately 260 µm, with a thickness starting at 23 µm at the base, decreasing to 19 µm at half-length, and culminating in a sharp 3 µm extremity at the top. The morphology of the cilium tip is further evidenced at higher magnification in Figure 2(3A,3B), revealing the formation of multiple lines of MP along the cilium length. Additionally, a cut cross-section of the cilium base is presented in Figure 2(4A,4B), offering detailed insight into how the particles are embedded in the PDMS matrix and distributed within the cilium bulk. This PDMS coverage offers valuable benefits by preventing particle leaching and minimizing corrosion resulting from exposure to biological fluids.

**Figure 3:**
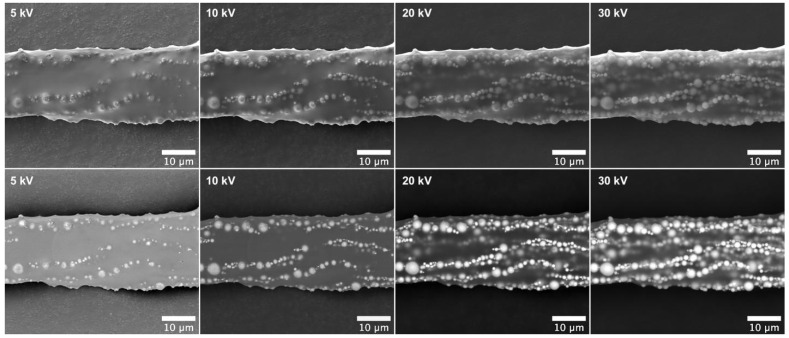
Scanning electron microscopy images of a cilium section were obtained with increasing voltages from 5 kV (**left**) to 30 kV (**right**). The upper images were obtained with an Everhart–Thornley detector (ETD) and the bottom images were obtained with a circular backscatter detector (CBS).

The distribution of the MPs within the cilium bulk is further evidenced in Figure 3. The series of SEM images, acquired at increasing voltages, reveals lines of MP present at different depths within the cilium body. By analyzing these images from left (5 kV) to right (30 kV), one can observe the distribution of MPs from closer to the surface down to the inner layers beneath, with the latter accessed using the more penetrating electron beam at 30 kV.

### 3.3. Fine-Tuning Cilia Dimensions

**Figure 4:**
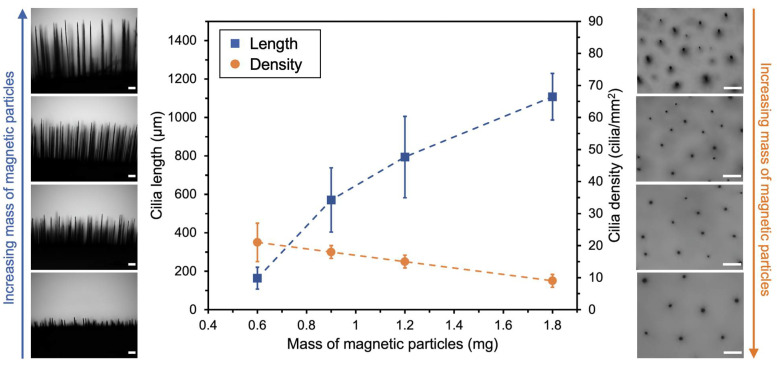
Variation of cilia length (**left**) and cilia density (**right**) as a function of the mass of magnetic particles used for cilia fabrication. The sample-to-magnet distance was kept constant at 1.2 cm. Error bars represent the combined standard deviation of two independent replicates considering the dimensions of at least 100 objects per sample. Optical microscopy images of the samples are presented on the left and right of the graph, showing lateral and top views, respectively. Scale bars equal 200 µm.

The dimensions of the fabricated cilia and their distribution over the surface are influenced by the applied magnetic field and the initial dispersion characteristics, such as nanoparticle concentration. Maintaining a constant magnetic field intensity allows for the enlargement of cilia dimensions (length and thickness) by increasing the concentration of the initial dispersion. Figure 4 illustrates potential variations in cilia fabrication by increasing the mass of magnetic particles (MP) from 0.6 mg to 1.8 mg in the initial dispersion while keeping the sample-to-magnet distance constant at 1.2 cm. Each data point represents the combined mean and standard deviation [29] of two independent replicates. In this case, the polymer concentration was adjusted accordingly to maintain a constant mass ratio of MP to PDMS at 1:10. When the mass of MP is tripled within this range, the average length of the resulting cilia increases nearly sevenfold, from 164 ± 57 µm to around 1100 ± 121 µm, and their thickness at half-length also increases from 26 ± 9 µm to 39 ± 10 µm. However, under these conditions, the number of cilia per surface area (cilia density) decreases by around 50%, from 21 ± 6 cilia/mm^2^ to 10 cilia ± 2 cilia/mm^2^, with the increase in MP mass. Notably, the greatest effect is observed in the cilia length, particularly at lower concentrations, where a 50% increase in MP mass results in a 250% increase in cilia length.

**Figure 5:**
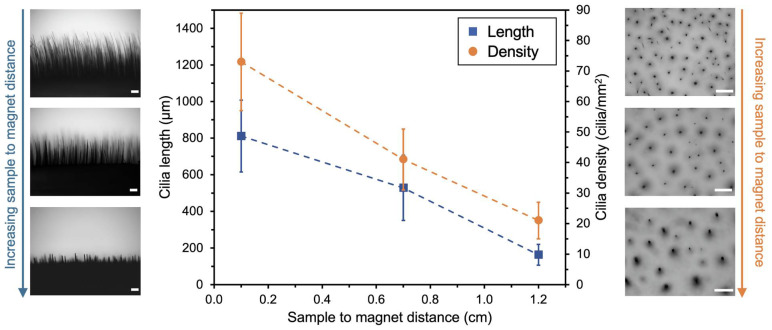
Variation of the cilia length (**left**) and cilia density (**right**) as a function of the sample-to-magnet distance during cilia fabrication. The mass of magnetic particles was kept constant at 0.6 mg. Error bars represent the combined standard deviation of two independent replicates considering the dimensions of at least 100 objects per sample. Optical microscopy images of the samples are presented on the left and right of the graph, showing lateral and top views, respectively. Scale bars equal 200 µm.

It is also possible to simultaneously increase the length of the cilia and the surface density while obtaining thinner cilia, as illustrated in Figure 5. This can be achieved by intensifying the effect of the magnetic field, for example, by approaching the sample to the magnet during preparation while keeping the mass of MP constant. By reducing the distance between the sample and the magnet from 1.2 to 0.1 cm during preparation, the average length of the resulting cilia increased nearly fivefold, from 164 ± 57 µm to 810 ± 196 µm, and the cilia density also increased approximately fourfold, from 21 ± 6 to 76 ± 16 cilia/mm^2^. However, the cilia become thinner when prepared under a more intense magnetic field, with their thickness at half-length varying from 26 ± 9 µm to 10 ± 4 µm within the analyzed range, resulting in average aspect ratios of up to 85. In this scenario, exposure to stronger magnetic fields closer to the magnet has the most pronounced impact on the cilia surface density. For instance, relocating the sample 0.6 cm away from the magnet resulted in a 50% reduction in cilia density. Conversely, at greater distances, the influence on the cilia length becomes more prominent.

**Figure 6:**
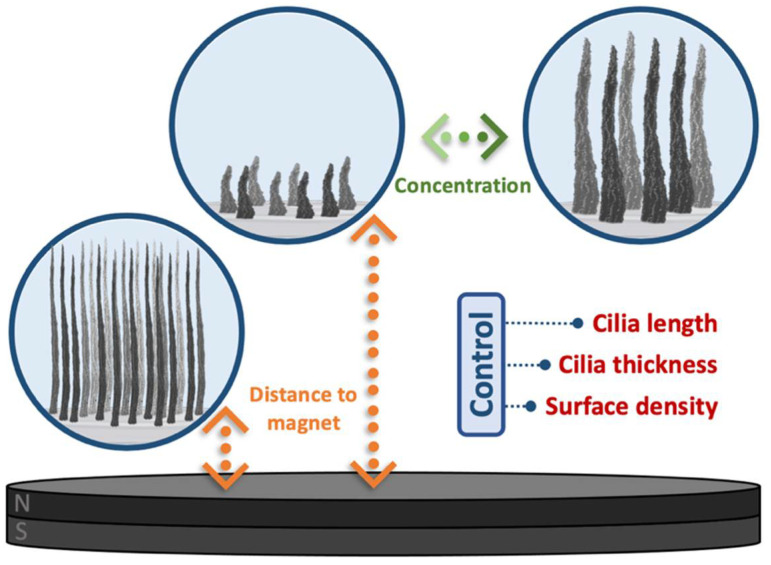
Illustrative diagram depicting the effects of sample-to-magnet distance and concentration of the initial dispersion during the fabrication process on resultant cilia characteristics, including length, thickness, and surface density.

The results highlight the remarkable flexibility of this preparation method in adjusting the dimensions of the cilia, as summarized in Figure 6. Following this protocol and fine-tuning two parameters independently, it is possible to adjust not only the cilia length (160–1100 µm) and thickness (10–40 µm at half-length), resulting in aspect ratios spanning from 6 up to 85, but also the number of cilia per surface area, ranging from 10 to approximately 80 cilia/mm^2^. Further combinations of these parameters can even increase these ranges. However, such flexibility inherently demands meticulous control and a consistent fabrication procedure to ensure a satisfactory reproducibility of the samples. Additional parameters, such as the magnetic particle-to-polymer ratio, dispersion volume, viscosity, magnetic particle size, or different mixture compositions can potentially influence the characteristics of the resulting cilia. These factors, as suggested in the literature, warrant further investigation to explore novel characteristics and possibilities [17,18,19,21,23].

### 3.4. Rationale for the Magnetic Cilia, and Qualitative Analysis of Their Interaction with Mucus

Mucus is a complex mixture of different secretions, forming a hydrophilic gel with viscoelastic properties. It covers nearly all of the entire airway epithelium, acting as a protective barrier against pathogens and air particulates. The mucus is continuously produced and secreted, and motility is facilitated by the ciliated cells lining the airway epithelial surface [33,34]. When the mucociliary clearance mechanism malfunctions, as seen in respiratory diseases, or when mechanically impaired by the presence of an indwelling silicone tracheal prosthesis, the mucus accumulates within the luminal aspect of the prosthesis. This organic material fosters the formation and fixation of a polymicrobial biofilm that interacts with the adjacent mucosa, leading to local tissue inflammation and, eventually, bacterial infections, delaying the tracheal healing process. Silicone prostheses (i.e., stents), such as the T-tube introduced in 1965 [35], and the straight studded stent introduced in 1990 [36], are endoscopically inserted into the airway lumen to provide support to the trachea, ensuring unobstructed breathing during tracheal disease resolution. Silicone stents are typically designed to remain in place for months to years but require periodic replacement due to silicone degradation and multimicrobial biofilm formation on their inner surfaces. The rationale for applying active magnetic cilia coupled to the lumen of the airway prosthesis is to facilitate unidirectional mucus movement out of the airway, potentially reducing biofilm adherence to the silicone surface and actively displacing it outward through a combination of metachronal motion and the cough mechanism. The anticipated outcome is a decrease in microbial colonization and luminal obstruction, prolonging stent lifespan and enhancing the patient’s quality of life by reducing the need for frequent stent changes.

**Figure 7:**
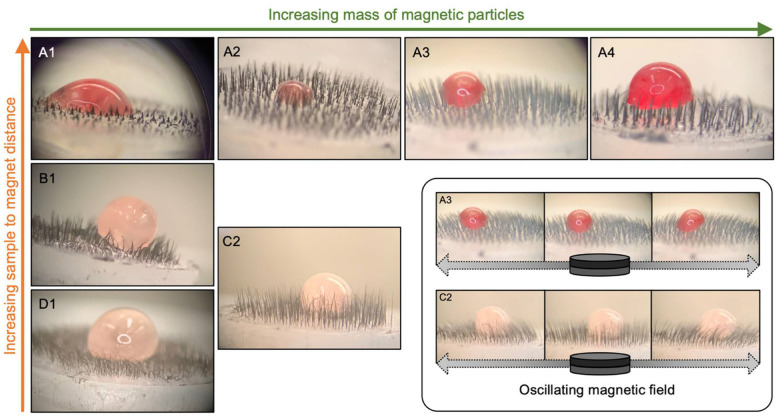
Optical microscopy images of cilia samples interacting with bullfrog mucus. Each image corresponds to a cilia sample prepared in a different condition combination. Images labeled 1 to 4 correspond to cilia prepared with increasing MP mass of 0.6, 0.9, 1.2, and 1.8 mg, respectively. Images labeled **A** to **D** correspond to cilia prepared under decreasing sample-to-magnet distances: 1.2, 0.7, 0.4, and 0.1 cm, respectively. The inset illustrates the motion of cilia samples **A3** and **C2** under an oscillating external magnetic field (refer to Videos S2 and S3 in the Supplementary Material).

The mucus produced by the bullfrog (*Rana catesbeiana*) exhibits viscoelastic properties similar to those of mammalian respiratory mucus, making it a standard model for mucociliary clearance studies [37,38,39]. Figure 7 presents optical microscopy images illustrating a qualitative study of the interaction between various magnetic cilia samples and the physiological mucus of *Rana catesbeiana*. We observed that longer cilia (A3 to D1) kept the mucus suspended for a period, preventing it from making close contact with the substrate surface. This was evident from the notably spherical shape of the mucus droplet, particularly observed for samples with densely packed cilia such as B1, D1, and C2. These samples exhibited a higher apparent contact angle (θ > 90°) with the interface, indicating lower wettability of the mucus on these surfaces. Conversely, shorter cilia (A1 and A2) were submerged by the mucus, which promptly spread over the substrate, resulting in an apparent contact angle below 90° with the interface. In all samples, the cilia structures mechanically withstood the addition of mucus, and thinner cilia presented a tendency to bend due to their higher flexibility. In addition, the structures retained their motion when stimulated with an external magnetic field, as illustrated in the highlighted input of Figure 7 and Videos S2 and S3 (Supplementary Material). In this study, the motion was induced only by the manual sliding of a magnet, and hence, it was not possible to observe the dislocation of the mucus. Further studies using a controlled magnetic field activation need to be performed to optimize the cilia motion conditions and generate an effective fluid flow [40]. Moreover, it was recently demonstrated that the presence of a hydrophilic lubricant layer within a ciliary array accelerates the transportation of viscous fluids, including porcine mucin, and thus, can be an interesting strategy to enhance mucociliary clearance in ciliated stents [41]. It is worth noting that the mucus deposition on the substrate can accelerate its dehydration and hinder its displacement through the coughing mechanism. Therefore, longer cilia structures, which could promote lower wettability and sustain mucus suspension, are likely better suited to facilitate mucus depuration and inhibit biofilm formation.

These preliminary results demonstrate the functionality of the magnetic active biomimetic cilia in actuating in a highly viscous medium, such as the airway mucus, potentially endowing silicone airway prosthesis with self-cleaning capabilities. This innovative concept, coupled with the method’s simplicity and affordability, can potentially revolutionize the design and functionality of silicone stents and other breathing devices, including orotracheal and tracheostomy cannulas, without incurring significant additional costs. Ongoing investigations are underway to further refine this fabrication approach, with a keen focus on optimizing the cilia metachronal motion under external control.

## 4. Conclusions

We present an original fabrication method to produce magnetic silicone cilia grafted to silicone surfaces and investigate their interaction with biological mucus, aiming to advance strategies for mitigating biofouling on silicone tracheal prostheses and enhance the functionality of these devices. Our approach, which combines magnetic self-assembly with polymer curing, offers remarkable adaptability, allowing for facile adjustments with minimal apparatus, facilitating the preparation of cilia with varying dimensions. By independently adjusting two fabrication parameters (mass of magnetic particles and sample-to-magnet distance), we achieved cilia with aspect ratios from 6 to 85, lengths spanning from 160 to 1100 µm, and surface densities from 10 to 80 cilia/mm^2^. These cilia structures exhibited robustness against the deposition of bullfrog mucus and maintained mobility within or beneath this viscoelastic fluid. Our preliminary investigation revealed distinct wettability profiles based on cilia dimensions, with longer cilia (>500 µm) and higher packing densities (>30 cilia/mm^2^) showing reduced mucus adhesion, suggesting their potential to facilitate mucus depuration. In summary, the present methodology offers simplicity and affordability for fabricating tailorable cilia suitable for diverse applications. The initial mucus studies highlight the mechanical resilience of the materials used, paving the way for further advancements aimed at improving the performance of silicone prostheses in clinical settings.

## Data Availability

The raw data will be made available by the authors on request.

## Supplementary Materials

The following supporting information can be downloaded at: https://www.mdpi.com/article/10.3390/bioengineering11030261/s1. Video S1. Magnetic susceptibility and flexibility demonstration of the magnetic cilia. Video S2. Motion of magnetic cilia with mucus. Reference of sample A3 in Figure 7. Video S3. Motion of magnetic cilia with mucus. Reference of sample C2 in Figure 7.
